# Life satisfaction: The role of domain‐specific reference points

**DOI:** 10.1002/hec.4412

**Published:** 2021-08-19

**Authors:** Sebastian Neumann‐Böhme, Arthur E. Attema, Werner B. F. Brouwer, Job N. J. A. van Exel

**Affiliations:** ^1^ Erasmus School of Health Policy & Management Erasmus University Rotterdam Rotterdam Netherlands; ^2^ Erasmus School of Economics Erasmus University Rotterdam Rotterdam Netherlands

**Keywords:** health, income, multiple discrepancy theory, reference points, subjective well‐being

## Abstract

In the evaluation of well‐being, it is not only important what people have in absolute terms, but also how this compares to reference points in relative terms. We explore the relevance of relative comparisons by testing the effect of people's self‐rated position on potential reference points for income and health on their subjective well‐being. We used Multiple Discrepancies Theory as a framework to identify seven potentially relevant reference points for income and health. A representative sample (*N* = 550) of the Netherlands assessed their income and health relative to these reference points. In addition, we elicited monthly household income, health status (EQ‐5D‐5L), and subjective well‐being (SWLS). In line with the literature, we found a negative convex relationship between subjective well‐being and age and a positive relationship with being employed, income, and health. For income, subjective well‐being was also associated with how current income compared to respondents' needs and progression over time, and for health especially with how current health compared to what they felt they deserved. Our findings suggest that income and health are important for subjective well‐being both in absolute and relative terms. We found negative effects on life satisfaction if some of the domain specific reference points were not met.

## 1 INTRODUCTION

There is increasing attention for subjective well‐being (SWB) in research (Diener et al., 1999; Dolan et al., 2008) and policy (Diener et al., 2010; Kahneman et al., 2004; OECD, 2017). SWB is a part of overall well‐being and is used as an umbrella term for how people think and feel about their life in general. More formally, “*SWB is defined as a person's cognitive and affective evaluations of his or her life*” (Diener et al., 2012). Previous research into the determinants of SWB suggested that income and health are two of the essential factors associated with SWB (Diener et al., 1999; Dolan et al., 2008). Nonetheless, how health and income attainments exactly translate into SWB is less obvious. Prospect theory (Kahneman & Tversky, 1979; Tversky & Kahneman, 1992) proposes that individuals use reference points when evaluating outcomes such as their health or wealth. Outcomes are processed as deviations from this reference point, often the status quo, and subsequently evaluated as a gain or a loss (Wakker, 2010). Several empirical studies showed that relative comparisons of income affect well‐being, for example, when it is compared to the income of others (Carlsson et al., 2007; Luttmer, 2005), or own past and prospective incomes (Easterlin, 2001). Although this suggests reference points are indeed relevant, it leaves open the question which reference points are used.

For most domains, it is unknown which ‐or indeed how many‐reference points people use. To avoid focusing on only one potential reference point, hence potentially missing relevant others, we apply Multiple Discrepancies Theory (MDT), developed by Michalos (1985). MDT suggests that multiple reference points could be used when evaluating one's situation, even simultaneously. A person's satisfaction with the situation is assumed to depend on perceived differences between what one has and seven points of comparison. These are what individuals think they need to survive (self‐needs), what individuals think they are entitled to (self‐deserve), what they would like to have (self‐wants), what people in their immediate environment have (self‐others), what individuals ever had before (self‐past), what they had expected to have now three years ago (self‐progress), and what they expect to have five years from now (self‐future).

The importance of income for SWB has been shown before (e.g. Lucas & Schimmack, 2009). Affluent individuals usually report a higher SWB than impoverished people. However, a more recent study by Jebb et al. (2018), building on the research of Kahneman and Deaton (2010), suggested that additional income only increases SWB up to a saturation point.

Based on US data, Easterlin (1980) observed that wealthier people reported a higher SWB, but that increases in the absolute income of a country did not raise overall SWB. This finding has been labelled the Easterlin paradox. When the study was repeated, including several industrialised and developing economies, Easterlin et al. (2010) found that short‐term happiness and income move in the same direction (e.g. downwards in a financial crisis). Nonetheless, in the long run (i.e., about 10 years), SWB does not increase together with the absolute income over time. One possible explanation for this paradoxical finding is that individuals compare their income to that of others and themselves in the past, which Clark et al. (2008) call internal and external reference points.

Previous studies found that not only absolute levels of income impact SWB (Diener et al., 1993) but also relative comparisons (Easterlin, 1980; Easterlin et al., 2010). For instance, Luttmer (2005) found that an increase in the neighbours' earnings negatively affects the well‐being of an individual. In related research, Kuhn et al. (2011) found that neighbours of lottery winners are more likely to (also) buy new cars, and Agarwal et al. (2018) showed that the magnitude of lottery prizes increased the number of subsequent bankruptcy filings of nonwinning peers in the neighbourhood. This suggests that relative comparisons can increase the consumption of peers, which is colloquially referred to as “keeping up with the Joneses” (Cambridge Dictionary, 2019).

The second domain considered to be one of the major determinants of SWB concerns health (Diener & Chan, 2011; Okun et al., 1984). The impact of health on SWB is substantial (Dolan et al., 2008), and the literature suggests the association between health and well‐being relies, at least in part, on relative comparisons (Diener et al., 1999). For example, results from the German Socio‐Economic Panel suggest that becoming sicker than the reference group worsens health satisfaction (Thiel, 2014). To a certain degree, there is interdependence between SWB and health and also income, where well‐being affects health and income. For example, Diener and Chan (2011) showed that SWB serves as a predictor of health and longevity, while for income, Oswald et al. (2008) find that people with higher well‐being are more productive.

The absolute health status of an individual tends to depreciate over time due to ageing. In theory, this would lead to an increasingly negative effect on SWB because of the deprecation of absolute health. This effect is offset by the process of adaptation, which mitigates the effect of declining health on SWB over time (Cubí‐Mollá et al., 2017; Etil et al., 2017; Powdthavee, 2009). An early example of adaptation concerns the case of extreme events, where a study by Brickman et al. (1978) compared the relative happiness of lottery winners and accident victims to a control group. They found that lottery winners were, in general, not happier than people in the control group because they gained less pleasure from ordinary events after experiencing an extremely positive event. In contrast, para‐ and quadriplegic accident victims reported their happiness at 2.96 on a five‐point scale within 12 months after their accident, compared to 1.28 shortly after the accident. In a study using the same dataset as the current study, Wouters et al. (2016) concluded that absolute improvements in health might improve SWB. However, perceived improvements relative to relevant reference points may have the same effect.

Overall, reference points and theories of reference dependence remain a theoretical and practical challenge in economics. On the theoretical side, Wakker (2010) argues that we still lack a comprehensive theory of how reference points are formed. Hence, both how and which reference points are formed is not easily theoretically predicted. Empirical studies may therefore provide information on which reference points are relevant in this context. The practical implications of existing multiple and domain‐specific reference points are relevant for policymakers who aim to maximise well‐being. To explain and understand how people react to policies or changes, we need to investigate which reference points are relevant for people's assessment of wellbeing. The reference points people use in such evaluations may be absolute (e.g. size of a payment) or relative (e.g. size of a payment relative to the one of others), and multiple, domain specific reference points may exist simultaneously. This may not only inform policies attempting to improve (relative) attainment, but also those trying to influence people's reference points themselves. Tversky and Kahneman (1981) for instance suggest that the framing of outcomes can shift a reference point and well‐being evaluations therefore may vary if an outcome is perceived as a gain or a loss. A better understanding of which reference points for income and health are associated with well‐being may therefore provide policymakers with valuable information on designing and framing their policies to maximise well‐being.

Hence, the objective of this paper is to explore the relevance of a broad range of potential reference points for income and health in the context of subjective well‐being.

## 2 METHODS

### 2.1 Subjects

The subject pool for the current study consisted of 550 respondents representative of the general public in the Netherlands between 18 and 75 years regarding age, gender, and education. A professional sampling company was hired to program the experiment and obtain the data through an Internet survey. Panel members received an invitation to participate in a web‐based questionnaire. By accepting the invitation to participate in this survey, respondents provided consent to use their responses for the purpose of this study. The data reported in this paper was collected in 2013 as part of a larger study. Here we only used a part of the relevant data for MDT and potential reference points; other elements were used for other purposes (Attema et al., 2018; Wouters et al., 2016). The relevant parts of the questionnaire can be found in the Supporting Information S1.

### 2.2 Design

The respondents first received questions regarding their sociodemographic characteristics. To account for respondents' personal characteristics, we selected age, gender, education level, marital status, having at least one child, and being unemployed from the sociodemographic section. For gender, we created a dummy variable taking the value 1 for males (49.3%) and 0 for females. We did the same for unemployment where the dummy took the value 1 if the respondent stated to be unemployed (11.8%) and 0 if they were working, retired, pensioners, doing housework, or were in training. A different dummy variable took the value 1 if respondents were married or living with a partner (59%) and the value 0 if they were single, divorced, widowers or in another type of relationship. Lastly, a dummy variable took the value of 1 if respondents had a least one child (57.5%) and 0 otherwise. Education was grouped into the categories low (28.6%), middle (41.6%) and high (29.8%).

In the second part of the survey, we asked subjects to complete the Satisfaction with Life Scale (SWLS), where they had to indicate to what extent they agreed with five propositions about their life (Diener et al., 1985). Each proposition consisted of seven response possibilities, varying from “completely disagree” to “completely agree”. The point values of the answers to the five propositions were then summarised into a composite well‐being index ranging from 7 (lowest possible well‐being) to 35 (highest possible well‐being). The third part of the survey was about income. Subjects were first asked to tick one of 13 income bands (see Appendix C in Supporting Information S1 for income bands used) in which their current monthly net household income fell. We used the midpoints of the chosen income band in the analyses; in case a subject picked the lowest [highest] band “below €999” [“€8000 or more”], they were asked to state their income in an open text field.

To elicit relative income positions, the net monthly household income stated before was displayed. We asked for (1) the income that would be sufficient for the respondents household to get by (*subsistence income;* see Appendix C in Supporting Information S1), and (2) the income that their household would need to be able to live a comfortable life without any worries (*luxury income;* see Appendix C in Supporting Information S1). These latter questions were specifically drawn up for this study, but resemble and build on previous work (Van Praag & Frijters, 1999). Individuals were asked to evaluate their income through a verbal qualifier such as sufficient or very good (here labelled as subsistence and luxury). As an alternative, externally determined measure of relative income, we used two reference budgets for households in the Netherlands (in 2014) that specified values for a “basic needs budget” and a “not‐much‐but‐adequate budget”, respectively (Hoff et al., 2016). These were coded as dummy variables taking the value of 1 if household income was below the household‐specific “basic needs budget” (24.7% of households in the sample) or “not‐much‐but‐adequate budget” (25.8% of households). We selected the “basic needs budget” as an externally determined measure of relative income in the regressions since this was conceptually closest to the subsistence income. Since the reference budgets for households in the Netherlands are based on the number of household members, we calculated separate budgets lines for each respondent based on their household composition.

Health state was elicited by using the EQ5D‐5L (Herdman et al., 2011) that captures the respondents' health states using five dimensions; that is mobility, self‐care, usual activities, pain/discomfort and anxiety/depression, each with 5 levels ranging from “1‐no problems” to “5‐extreme problems”. We added a sixth dimension to the questionnaire to better account for neuropsychiatric health problems. This question captured cognitive health or functioning, for example memory, concentration, coherence, and IQ. A more detailed discussion of why this is a relevant addition to the EQ5D‐5L can be found elsewhere (Wouters et al., 2016). Based on these absolute health assessments, we generated a health problem index by summarising the scores of the EQ5D‐5L and the cognitive dimension, which therefore ranges from 6 (best health) to 30 (worst health).

### 2.3 Reference points for income and health

Finally, the MDT dimensions cover a broader range of relative comparisons. To cover these, we asked respondents to compare their current income to a set of seven reference points derived from MDT. These are what individuals think they need to survive (self‐needs), what individuals think they are entitled to (self‐deserve), what they would like to have (self‐wants), what people in their immediate environment have (self‐others), what individuals ever had before (self‐past), what they had expected to have now three years ago (self‐progress), and what they expect to have five years from now (self‐future).

Each comparison could be rated on a nine‐point Likert scale ranging from “not good at all” (1) over “neutral” (5) to “very good” (9). The set of questions can be found in Appendices A and B (see Supporting Information S1). Based on MDT, life satisfaction may depend on comparisons between the current conditions (here in terms of income and health) and the standards described in MDT. A discrepancy resulting from an upward comparison (i.e. the standard is higher than the current situation) is expected to have a negative impact on satisfaction, whereas a downward comparison (current situation is better than the standard) is expected to have a positive impact (Michalos, 1985; Diener et al., 1999). We expect a more substantial impact on well‐being from downward comparisons since prospect theory predicts that losses loom larger than gains of the same magnitude (Kahneman & Tversky, 1979). Therefore, we generated dummy variables for each MDT item that takes the value of 1 for an upward comparison, that is if the rating is anywhere between “not good at all” and “not good” (1–4 on the nine point Likert scale) and 0 for neutral to very good (5–9). With this approach, we take neutral (5) as the status quo and anything below neutral as a loss compared to the offered reference point, for example what individuals think they need to survive (self‐needs). Consequently, we expect a negative association with the SWLS score for all MDT reference point dummy variables.

### 2.4 Data analysis

Descriptive statistics of the sample and the MDT domains were first derived. Next, we employed independent‐samples *t*‐tests to compare well‐being of those being below neutral to those above neutral in each of the MDT domains. Furthermore, we looked at the distribution of the sample across the relative income categories and the corresponding well‐being ratings. Since one might argue that the MDT domains elicit quite similar aspects (e.g. self‐future and self‐progress) regarding income and health, we inspected the correlation between the MDT domains. To that end, we used Spearman's rank correlation coefficients to assess the relationships between the MDT reference points in both domains. We proceeded by using the Variance Inflation Factor (VIF) to measure the degree of multicollinearity. A VIF is commonly seen as unproblematic if it is below 10 (Chatterjee et al., 2000; Neter et al., 1990).

Finally, we employed a stepwise ordinary least squares regression approach to investigate the relationship between SWB and the reference points for income and health. We estimated a total of 8 models, each testing variables of interest or alternative specifications, for example for different measures of relative income in model II and model III. More specifically, model I was the basic model, including only sociodemographic characteristics, absolute income and health, the latter measured by the health problem index. We included the dummy variables signalling unemployment, being married or living together and having at least one child. Furthermore, we added age squared and the health problem index squared to test for a non‐linear relationship between these variables and SWB.

Model II added the relative income variables displayed in Table 1 to Model I, as a dummy variable that is equal to 1 for respondents with an income higher than their subsistence level, or at least as high as their luxury income level (i.e., C, D and E in Table 1), and 0 otherwise. Having an income below or at the subsistence level served as a base. Model III tested the alternative specification of relative income, being below the “basic needs budget”, as an alternative to the subsistence and luxury income levels. Model IV added the MDT reference points for the income domain to Model I. Model V tested the self‐stated relative incomes levels combined with the MDT income reference points. In Model VI, we tested the MDT reference points for health in the basic specification and added relative income (subsistence and luxury level income) in Model VII. Model VIII ultimately included all potential reference points for income and health. This stepwise approach aimed to provide tractable insights into the influence of specific factors, such as relative income and showed the robustness of estimates.

**TABLE 1:** Distribution of relative income categories and corresponding subjective well‐being (SWLS) values

Variable	SWLS Compact				
	Obsv.	Mean	sd	Min	Max
A: Has income below their own subsistence level	214	20.97	7.33	5	35
B: Has income at subsistence level (below luxury level)	108	24.26	5.67	8	35
C: Has income in‐between subsistence and luxury level	112	26.14	5.53	9	35
D: Has income at luxury level (above subsistence level)	82	27.51	5.42	10	35
E: Has income at both subsistence and luxury level (all 3 values are the same)	34	24.15	7.64	5	35
Sum	550	23.84	6.89	5	35

## 3 RESULTS

Table 2 presents the sample characteristics, while Table 3 presents descriptive statistics of the MDT reference points for income and health. Mean net household income was € 2152.27 (median: 1750, SD: 1310.29, IQR = 1500), mean subsistence income was € 2080 (median: 1750, SD: 1204.67, IQR = 1000) and median luxury income was € 2750, with the mean € 185,518 (SD: 4,263,867, IQR = 1500) being skewed due to a few outliers in the data.

**TABLE 2:** Descriptive summary statistics of demographic and health characteristics of the sample

Variable	Level	Sample Statistic
Age (S.E., range)		45.6 (0.64, 18–75)
Gender (% male)		49.3
Education	Low	28.6
	Medium	41.6
	High	29.8
Employment status	Employed	48.1
	Unemployed	11.8
	Other	40.1
Household income	Low (<1500 €)	33.8
	Middle (1500 € ‐ 3999 €)	59.5
	High (>3999 €)	6.7
Marital status	Married or living together	59.1
	Other	40.9
Children	Yes	57.5
	No	42.5
Health	EQVAS (S.E., range)	76.8 (0.76, 9–100)
	Mobility problems	28.0
	Self‐care problems	7.5
	Usual activities problems	30.0
	Pain/discomfort problems	56.9
	Anxiety/depression problems	27.6
	Cognitive functioning problems	22.4

**TABLE 3:** Descriptive statistics of the MDT domains

Reference Points	Mean	S.E. and Range	Mean	S.E. and Range
	Income domain		Health domain	
Self‐needs	5.9	(0.09, 1–9)	6.6	(0.07, 1–9)
Self‐deserves	5.6	(0.09, 1–9)	6.4	(0.07, 1–9)
Self‐wants	5.4	(0.09, 1–9)	6.7	(0.08, 1–9)
Self‐others	5.5	(0.08, 1–9)	6.4	(0.07, 1–9)
Self‐past	5.7	(0.09, 1–9)	6.5	(0.09, 1–9)
Self‐progress	5.4	(0.09, 1–9)	6.3	(0.08, 1–9)
Self‐future	5.5	(0.09, 1–9)	6.5	(0.08, 1–9)

The distribution of the respondents in terms of the relative income categories and the mean SWLS scores associated with them are presented in Table 1. Some participants seemed to have struggled with the relative income questions, as indicated by category E. Characteristic for these 34 participants, who reported the same value for subsistence and luxury income, was that they spent significantly less time with the questionnaire than all other participants (others gave the same score for all MDT questions). As a robustness check, the analysis was repeated, excluding 48 participants that showed the similarities described above (category E in Table 1 and MDT in patterns), but this did not change the results.

**FIGURE 1:**
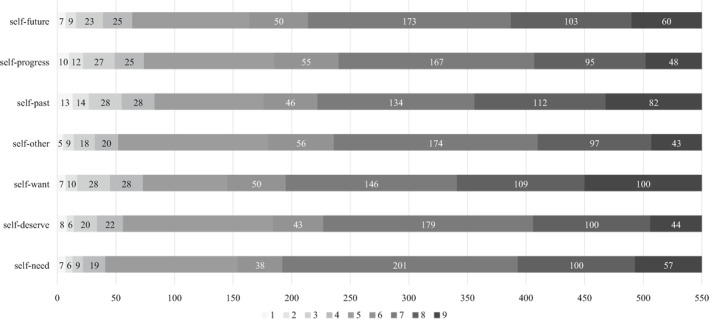
Distribution of responses in the multiple discrepancies theory Domains for income

**FIGURE 2:**
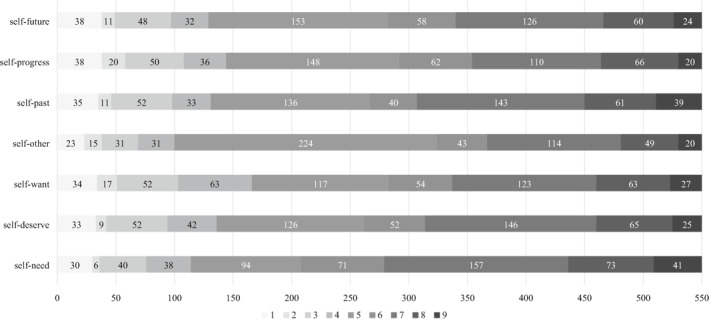
Distribution of responses in the multiple discrepancies theory Domains for health

Given the descriptive statistics, we inspected the correlation between the MDT domains for income and health. The answer patterns are reported in Figures 1 and 2. Furthermore, Spearman's rank correlation coefficients matrices are reported in Tables S1 and S2 (see Appendix D in Supporting Information S1) to assess the relationships between the MDT reference points in both domains. The results suggest a positive correlation between MDT reference points in both the health and income domain. The MDT domains' VIF ranged from 1.54 to 2.67, which suggests an unproblematic degree of collinearity in the regressions (Chatterjee et al., 2000; Neter et al., 1990).

The results from the independent‐samples *t*‐test in Table 4 established a statistically significant difference in SWB values for being below or above the reference point (neutral) across the MDT reference points for health and income. We furthermore observed differences in SWLS scores in the relative income categories A‐D. This may suggest that living below what oneself considers her or his subsistence level income was associated with lower SWB. On the other end of the spectrum, having an income at or above what respondents considered their luxury level income was associated with higher SWB, which intuitively makes sense.

**TABLE 4:** Distribution of MDT reference points for income and health and corresponding subjective well‐being (SWLS) values using independent sample *t*‐tests

		SWLS Compact for Income				SWLS Compact for Health			
MDT Domain	Level	Obs.	Mean	sd	*p*‐value	Obs.	Mean	sd	*p*‐value
Self‐need	Neutral or above	436	25.47	5.75	0.0000	509	25.42	6.33	0.0000
	Below neutral	114	17.62	7.36		41	15.44	7.95	
Self‐deserve	Neutral or above	414	25.33	5.98	0.0000	494	24.82	6.17	0.0000
	Below neutral	136	19.32	7.50		56	15.20	6.89	
Self‐want	Neutral or above	384	25.59	5.93	0.0000	477	24.70	6.35	0.0000
	Below neutral	166	19.79	7.27		73	18.21	7.65	
Self‐others	Neutral or above	450	19.9	7.60	0.0000	498	24.44	6.52	0.0000
	Below neutral	100	24.72	6.41		52	18.12	7.72	
Self‐past	Neutral or above	419	25.26	6.23	0.0000	467	24.78	6.34	0.0000
	Below neutral	131	19.29	6.94		83	18.53	7.49	
Self‐progress	Neutral or above	406	25.56	5.98	0.0000	476	24.80	6.34	0.0000
	Below neutral	144	19.00	7.00		74	17.69	7.17	
Self‐future	Neutral or above	421	25.23	6.07	0.0000	486	24.83	6.34	0.0000
	Below neutral	129	19.31	7.47		64	16.33	6.27	

Table 5 reports the results of the eight regression models (robust standard errors in Table 6). Across models, SWB was decreasing up to age 40‐45 and then increasing. Furthermore, in line with expectations, well‐being was positively associated with income and negatively with unemployment. The association of SWB with the health problems index was also convex, indicating that more health problems were associated with lower SWB, but at a decreasing rate. Both living together and having children were positively associated with SWLS scores, but only at the 10% level and not consistently throughout models.

**TABLE 5:** OLS regression of SWLS on respondent characteristics, health, and MDT for monetary and health questions

SWLS	Model I	Model II	Model III	Model IV	Model V	Model VI	Model VII	Model VIII
Age	−0.27**	−0.25**	−0.26**	−0.19*	−0.18*	−0.30***	−0.29***	−0.23**
Age^2^	0.0031***	0.0029**	0.0031***	0.0023**	0.0022**	0.0034***	0.0032***	0.0026**
Male (base: female)	−0.80	−1.06**	−0.76	−0.91*	−1.06**	−0.22	−0.51	−0.66
Education level (base: low)								
Middle	0.78	0.54	0.82	1.12*	0.96*	0.60	0.39	0.74
High	0.85	0.54	0.79	1.37**	1.14*	0.72	0.44	0.90
Unemployed (base: working/training/retired)	−3.10****	−3.13****	−3.15****	−1.68**	−1.78**	−2.97****	−2.99****	−1.93**
Married or living together (base: single, divorced, widow, other)	1.09*	1.13*	1.04	0.79	0.82	0.97*	1.01*	0.79
Has at least one child (base: no children)	0.64	0.92	0.61	0.75	0.93	0.76	1.01*	1.03*
Income (base: low <1500)								
Middle income (1500‐4000)	1.09*	0.11	1.23	0.049	−0.47	1.08*	0.20	−0.30
High income (>4000)	3.69****	1.53	3.85***	2.17**	0.83	3.08***	1.19	0.75
Relative income (base: income below or at subs. Level)								
Income between subs. & luxury level		2.20****			1.19**		2.02****	1.26**
Income at or above luxury level		3.38****			2.32***		3.00****	2.26***
Income < basic needs budget			0.20					
Health problem index	−1.85****	−1.73****	−1.89****	−1.47****	−1.43****	−1.40****	−1.30****	−1.21****
Health problem index^2	0.039**	0.035**	0.040**	0.030**	0.029*	0.034***	0.030**	0.027**
MDT reference points for income (base: better than neutral)								
Self‐need				−2.95****	−2.80***			−2.31***
Self‐deserve				−0.91	−0.82			−0.46
Self‐want				−0.79	−0.69			−0.68
Self‐others				0.24	0.27			0.11
Self‐past				−0.82	−0.91			−0.74
Self‐progress				−2.29***	−2.23***			−1.97**
Self‐future				0.51	0.66			0.79
MDT reference points for health (base: better than neutral)								
Self‐need						−2.20	−2.11	−1.77
Self‐deserve						−3.62****	−3.43****	−2.27**
Self‐want						1.29	0.99	1.60
Self‐others						−1.42	−1.50	−1.51*
Self‐past						−0.71	−0.41	−0.36
Self‐progress						−1.87*	−1.69*	−1.22
Self‐future						−1.44	−1.58	−1.39
R‐squared	0.334	0.361	0.335	0.439	0.450	0.412	0.434	0.486

****p < 0.001.

***p < 0.01.

**p < 0.05.

*p < 0.10.

**TABLE 6:** Robust standard errors for all models in parentheses

SWLS	SE MI	SE M II	SE M III	SE M IV	SE M V	SE M VI	SE M VII	SE M VIII
Age	(0.11)	(0.11)	(0.11)	(0.099)	(0.098)	(0.10)	(0.100)	(0.096)
Age^2^	(0.0012)	(0.0011)	(0.0012)	(0.0011)	(0.0011)	(0.0011)	(0.0011)	(0.0010)
Male (base: female)	(0.50)	(0.50)	(0.51)	(0.47)	(0.46)	(0.49)	(0.48)	(0.46)
Education level (base: low)								
Middle	(0.64)	(0.63)	(0.64)	(0.58)	(0.58)	(0.59)	(0.59)	(0.56)
High	(0.68)	(0.68)	(0.69)	(0.63)	(0.64)	(0.66)	(0.65)	(0.62)
Unemployed (base: working/training/retired)	(0.88)	(0.87)	(0.91)	(0.77)	(0.77)	(0.84)	(0.84)	(0.79)
Married or living together (base: single, divorced, widow, other)	(0.64)	(0.63)	(0.66)	(0.62)	(0.62)	(0.58)	(0.58)	(0.58)
Has at least one child	(0.65)	(0.63)	(0.66)	(0.60)	(0.59)	(0.61)	(0.60)	(0.58)
Income (base: low <1500)								
Middle income (1500‐4000)	(0.62)	(0.63)	(0.98)	(0.60)	(0.60)	(0.58)	(0.59)	(0.59)
High income (>4000)	(1.08)	(1.13)	(1.37)	(1.01)	(1.05)	(1.01)	(1.04)	(1.00)
Relative income (base: income below or at subs. Level)								
Income between subs. & luxury level		(0.59)			(0.57)		(0.57)	(0.57)
Income at or above luxury level		(0.72)			(0.71)		(0.68)	(0.68)
Income < basic needs budget			(1.01)					
Health problem index	(0.39)	(0.39)	(0.39)	(0.36)	(0.37)	(0.33)	(0.33)	(0.33)
Health problem index^2	(0.016)	(0.016)	(0.016)	(0.015)	(0.015)	(0.013)	(0.013)	(0.013)
MDT reference points for income (base: better than neutral)								
Self‐need				(0.88)	(0.87)			(0.89)
Self‐deserve				(0.87)	(0.86)			(0.84)
Self‐want				(0.72)	(0.72)			(0.72)
Self‐others				(0.81)	(0.81)			(0.78)
Self‐past				(0.72)	(0.70)			(0.69)
Self‐progress				(0.80)	(0.79)			(0.80)
Self‐future				(0.79)	(0.78)			(0.81)
MDT reference points for health (base: better than neutral)								
Self‐need						(1.35)	(1.30)	(1.22)
Self‐deserve						(0.98)	(0.95)	(0.93)
Self‐want						(1.04)	(1.03)	(1.03)
Self‐others						(0.96)	(0.95)	(0.90)
Self‐past						(1.05)	(1.05)	(1.04)
Self‐progress						(1.04)	(1.01)	(1.00)
Self‐future						(1.06)	(1.02)	(0.98)

The relative income expressed by having an income above the stated subsistence and luxury income introduced in model II (also part of model V and VII‐VIII) was associated with a significantly higher SWB compared to people at or below the subsistence level. The alternative (external) specification of relative income, having an income below the “basic needs budget”, was not significant. Once the subsistence and luxury income dummies were added, absolute income was no longer significant, suggesting relative income may be more strongly associated with SWLS scores than absolute income.

When applying the MDT reference points for income in model IV‐V and model VIII, we identified that two MDT reference points, namely self‐need and self‐progress, were significantly associated with SWB. As expected, the signs were negative, meaning that having an income in the loss domain (worse than neutral) compared to the income one would need to survive and what respondents had expected to have at that point in time was associated with a negative impact on SWB.

We investigated the effect of the MDT reference points for health in model VI without relative income and in model VII, including it. In the health domain, self‐deserve was highly significant with a negative sign throughout models VI‐VIII, implying that having a health status that is considered worse than what respondents believed they are entitled to was associated with a lower SWLS score. We identified two more potential reference points in the health domain, but not consistently throughout models and only at the 10% level. Self‐progress, rating own health worse than the health the respondent expected to have, was only significant in models where we included reference points for health (model VI & VII), but not when combined with the MDT reference points for income. When these were added (Model VIII), self‐others was significant. This suggests that when comparing the own health to that of others in the immediate environment, a health state seen as worse than neutral was associated with a lower SWB.

Overall, our results indicate that in the income domain, SWB was primarily associated with people's comparison to their needs and their progression over time compared to expectations about this in the past. In the health domain, what people think they deserve was significantly associated with SWB, whereas the association with other reference points was less clear. Furthermore, as compared to health, relative income variables appeared comparatively more relevant than absolute income variables for SWB. For health, the addition of the MDT reference points was relevant in explaining differences in SWB. Still, it did not affect the relevance of absolute health, expressed by the health problem index.

## 4 DISCUSSION

There is a growing interest in how people evaluate outcomes relative to reference points. Here, we investigated the relationship between subjective well‐being and multiple potential reference points for income and health derived from multiple discrepancies theory (Michalos, 1985). This provided insights into which reference points respondents may use when evaluating their subjective well‐being. To our knowledge, this is the first application of MDT to identify which potential reference points people apply in the income and health domains, which are seen as two of the most important contributors to subjective well‐being (van Praag et al., 2003). Our results allowed us to identify to which reference points individuals compared their income and health in assessing SWB. This may help to further understand SWB assessments and future research in identifying how exactly reference points are formed and why.

Our results suggest that in our sample, multiple reference points were associated with life satisfaction. We found significant negative effects on life satisfaction measured by the SWLS when there were discrepancies between the current income and the perceived self‐need or self‐progress. An upward comparison, meaning that the current income was rated worse than what respondents think they need or expected to have, was associated with lower satisfaction. This finding is intuitive and in line with the theory (Diener et al, 1999; Michalos, 1985). Falling short of an expectation or reference level would be expected to have a negative impact on SWB. Indeed, Stutzer (2004) also highlights the importance of relative income and suggests that subjective well‐being depends on gaps between the actual income and aspirations rather than on absolute income levels.

Similarly, we found that relative income (i.e. actual income compared to self‐stated subsistence and luxury incomes) was significantly associated with SWB throughout all models. In contrast, the comparison to the basic budget line, which could be seen as an external relative income category, was not significantly associated with SWB. Furthermore, the basic budget line values, relevant in Dutch policy, were substantially below what respondents believed to be a subsistence income. For example, median subsistence household income for couples with no children was € 2250 as indicated by themselves, while, in contrast, the basic budget for this household would be € 1330 (Hoff et al., 2016). This suggests that (self‐perceived) relative income may be more relevant for SWB than absolute income and that own assessments of minimum levels of income may be more important than ‘objective’ figures. Other studies (Gabillard & Duesenberry, 1953; Luttmer, 2005) suggested that relative income in the form of social comparisons is important for subjective well‐being, which we could not confirm in our study. Differences in income inequality between countries, which are lower in the Netherlands than, for instance, in the United States or the United Kingdom (OECD, 2019; van Doorslaer & Koolman, 2004), may partly explain this.

In the health domain, being in a health state that was perceived as worse than what respondents thought they deserved was highly significantly and negatively associated with life satisfaction measured by the SWLS, similar to findings by Wouters et al. (2016). In agreement with Graham et al. (2011), we found that comparing own health to that of people in one's direct environment (self‐others) was significantly associated with life satisfaction. Graham et al. (2011) found a positive impact of reference group health in a Latin American context. In contrast, we found that if the own health state was considered worse than that of the reference group's health, this was negatively associated with SWB in our sample from the Netherlands. However, this association was only marginally significant and was not consistently observed throughout different models, for example not when only the MDT reference points for health were included (regression models VI‐VII). One further reference point for health, self‐progress, also was sometimes found to be potentially relevant. Our findings in this sense partly resemble those of Michalos (1991, 2004), who found that gaps in self‐wants and self‐others had the most considerable impact on health satisfaction in a large cross‐country student sample. Our findings suggest that reference points are associated with SWB scores in both the income and health domain, but different reference points may be relevant in these domains and further research may explore these reference points further.

Some limitations of this study need noting. First, we emphasise that we use the term ‘reference points’ somewhat loosely compared to its definition in prospect theory. Indeed, we did not precisely quantify the income or health levels below which an individual would consider him‐ or herself in the loss domain, nor did we directly observe inflexion points below and above these points. We instead investigated which types of reference points within the set provided by MDT were significantly associated with SWB, which is merely suggestive of functioning as reference points.

Second, the dataset used for this research was obtained in a representative sample of the Dutch population. Therefore, the generalisability of our results to other settings (e.g. countries with lower levels of income and health or more inequality) is limited. Repeating this study in larger samples would be of value as well. This might also allow a closer investigation of SWB changes on the Likert scale.

Third, this study only identified associations between reference points in the respective domains and subjective well‐being, limiting its contribution. Future research could use more formal tests to investigate whether the here identified potential reference points actually serve as formal reference points, for instance using an experimental design (e.g. Lipman et al., 2020). Causal relationships could also be investigated using panel data by investigating the effect of changes in variables serving as reference points.

Fourth, we used an unweighted composite index of the five propositions in the Satisfaction with Life Scale in this study. The advantage of such a multi‐item scale is that it has higher reliability and is less prone to differences in scale interpretation across individuals than single‐item scales (OECD, 2013). On the other hand, Cheung and Lucas (2014) showed that single‐item scales can perform as good as the multi‐item SWLS when eliciting life satisfaction. Since there is no established standard to measure life satisfaction (OECD, 2013), we summarised the result in one index. Using a single‐item scale or a composite index out of both (Himmler et al., 2020) would also have been options. Some authors argue that the last two items of the SWLS should be omitted (Lamu & Olsen, 2018) since these two items are age‐sensitive (Zou et al., 2013). We tested this approach as a sensitivity analysis, but it did not change our results.

Furthermore, there were some incoherent answers by respondents, such as answering in patterns or stating illogical relative incomes such as a luxury income below the subsistence income. This may be due to the length of the overall questionnaire or a problem with the relative income categories. We conducted a sensitivity analysis to address this potential issue, excluding all participants that showed incoherent answers (*n* = 182). The results of the sensitivity analysis were similar to model VIII. The directions of the coefficients did not change, but the self‐others reference point for health was no longer statistically significant.

Fifth, we only focused on income and health as two essential domains in determining overall well‐being using self‐reported values. Future research into identifying reference points in other domains and the quantification of these and those highlighted in this paper is encouraged.

Overall, our results suggest that people may use multiple and domain‐specific reference points when evaluating SWB. This would imply that people focus on multiple and domain‐dependent comparisons when evaluating their achievements in specific domains to determine subjective well‐being. Furthermore, our results imply that money matters, but primarily in relative terms. SWB appears to be substantially negatively affected when current income is below what people think they need to ‘survive’ (self‐need), although we only observed associations. One could claim that Dutch social security legislation already incorporates this critical aspect, given that unemployment benefits are relative to the last earned income (European Commission, 2018).

In addition, policymakers could use framing to influence people to adopt specific reference points (Tversky & Kahneman, 1981). For example, government payments to mitigate the effect of an economic crisis could be framed to enable recipients to meet their self‐needs. If respondents took this reference point instead of a self‐past one, this could improve both SWB and support for the policy.

In conclusion, people appear to have multiple and domain‐specific reference points to compare their current achievements when assessing SWB. The smaller the discrepancy between what they have and what they think they need or deserve, the higher the well‐being they experience. Reference points can relate to absolute levels of endowment, but also to relative ones, and the degree to which these matter also appears to be domain‐specific.

## CONFLICT OF INTEREST

The author declares that there is no conflict of interest.

## Supporting information

Supplementary Material

## Data Availability

The data that support the findings of this study are available from the corresponding author upon reasonable request.
