# Supplementary material for: Life satisfaction: The role of domain‐specific reference points
Source: Health Econ. 2021 Aug 19;30(11):2766–79. doi: 10.1002/hec.4412 (PMC9291216; doi:10.1002/hec.4412)
Supplement: Supplementary file 1 — Supplementary Material [file HEC-30-2766-s001.docx]

# Appendix

## Appendix A: Reference points for health question

How do you rate your health compared to ...

|  | Not good at all |  | Not Good |  | Neutral |  | Good |  | Very Good |
| --- | --- | --- | --- | --- | --- | --- | --- | --- | --- |
| 1. what you need to survive | 1 | 2 | 3 | 4 | 5 | 6 | 7 | 8 | 9 |
| 1. what you think you are entitled to | 1 | 2 | 3 | 4 | 5 | 6 | 7 | 8 | 9 |
| 1. how healthy you would like to be | 1 | 2 | 3 | 4 | 5 | 6 | 7 | 8 | 9 |
| 1. how healthy people are in your immediate environment (e.g. friends, colleagues, neighbors) | 1 | 2 | 3 | 4 | 5 | 6 | 7 | 8 | 9 |
| 1. how healthy you have ever been before | 1 | 2 | 3 | 4 | 5 | 6 | 7 | 8 | 9 |
| 1. how healthy you expected to be now three years ago | 1 | 2 | 3 | 4 | 5 | 6 | 7 | 8 | 9 |
| 1. how healthy you expect to be in 5 years | 1 | 2 | 3 | 4 | 5 | 6 | 7 | 8 | 9 |

## Appendix B: Reference points for income question

How do you rate your income compared to ...

|  | Not good at all |  | Not Good |  | Neutral |  | Good |  | Very Good |
| --- | --- | --- | --- | --- | --- | --- | --- | --- | --- |
| 1. what you need to survive | 1 | 2 | 3 | 4 | 5 | 6 | 7 | 8 | 9 |
| 1. what you think you are entitled to | 1 | 2 | 3 | 4 | 5 | 6 | 7 | 8 | 9 |
| 1. what you would like to earn | 1 | 2 | 3 | 4 | 5 | 6 | 7 | 8 | 9 |
| 1. what people in your immediate vicinity (e.g. friends, colleagues) make | 1 | 2 | 3 | 4 | 5 | 6 | 7 | 8 | 9 |
| 1. what you have ever earned before | 1 | 2 | 3 | 4 | 5 | 6 | 7 | 8 | 9 |
| 1. what you expected to earn today three years ago | 1 | 2 | 3 | 4 | 5 | 6 | 7 | 8 | 9 |
| 1. what you expect to earn in 5 years | 1 | 2 | 3 | 4 | 5 | 6 | 7 | 8 | 9 |

## Appendix C: Subsistence and luxury level income questions

*Subsistence level income*

You indicated earlier that the net income of your household is about € NOWINC per month. Which net income per month would be sufficient for your household to just get by? Assume that you continue to live in your current home, but otherwise you live very economically, do not save and do not go on vacation.

- below 999
- 1000-1499
- 1500-1999
- 2000-2499
- 2500-2999
- 3000-3499
- 3500-3999
- 4000-4499
- 4500-4999
- 5000-5999
- 6000-6999
- 7000-7999
- 8000 or more

*Luxury level income*

You indicated earlier that the net income of your household is about € NOWINC per month. What net income per month would be enough for your household to live very well, without financial worries? Assume here that you can do everything you want, but without giving large amounts to others.

- below 999
- 1000-1499
- 1500-1999
- 2000-2499
- 2500-2999
- 3000-3499
- 3500-3999
- 4000-4499
- 4500-4999
- 5000-5999
- 6000-6999
- 7000-7999
- 8000 or more

## Appendix D: Correlation Matrix

Table 6: Spearman rank correlation matrix of the health-related MDT domains (* p<0.01).

| Domain |  | Health | Health | Health | Health | Health | Health | Health |
| --- | --- | --- | --- | --- | --- | --- | --- | --- |
|  | MDT | Need | Deserve | Wants | Others | Past | Progress | Future |
| Health | Need | 1 |  |  |  |  |  |  |
| Health | Deserve | 0,6911* | 1 |  |  |  |  |  |
| Health | Wants | 0,5578* | 0,5691* | 1 |  |  |  |  |
| Health | Others | 0,5202* | 0,4965* | 0,6722* | 1 |  |  |  |
| Health | Past | 0,5202* | 0,4594* | 0,6617* | 0,5752* | 1 |  |  |
| Health | Progress | 0,4658* | 0,5592* | 0,6323* | 0,6055* | 0,6712* | 1 |  |
| Health | Future | 0,5835* | 0,6040* | 0,6121* | 0,5378* | 0,5402* | 0,7412* | 1 |

Table 7: Spearman rank correlation matrix of the income-related MDT domains (* p<0.01).

| Domain |  | Income | Income | Income | Income | Income | Income | Income |
| --- | --- | --- | --- | --- | --- | --- | --- | --- |
|  | MDT | Need | Deserve | Wants | Others | Past | Progress | Future |
| Income | Need | 1 |  |  |  |  |  |  |
| Income | Deserve | 0,7247* | 1 |  |  |  |  |  |
| Income | Wants | 0,6676* | 0,7882* | 1 |  |  |  |  |
| Income | Others | 0,5736* | 0,6078* | 0,6253* | 1 |  |  |  |
| Income | Past | 0,6183* | 0,6570* | 0,6919* | 0,6134* | 1 |  |  |
| Income | Progress | 0,6622* | 0,7093* | 0,7274* | 0,6327* | 0,7886* | 1 |  |
| Income | Future | 0,6581* | 0,6764* | 0,6886* | 0,6030* | 0,6525* | 0,8073* | 1 |

| **Strength of the**  **correlation** (Evans, 1996) | | | |
| --- | --- | --- | --- |
|  | 00-.19 “very weak” |  |  |
|  | .20-.39 “weak” | |  |
|  | .40-.59 "moderate" | | |
|  | .60-.79 "strong" | |  |
|  | .80-1.0 "very strong" | | |
